# Models of autoimmune demyelination in the central nervous system: on the way to translational medicine

**DOI:** 10.1186/2040-7378-1-5

**Published:** 2009-10-21

**Authors:** Ralf A Linker, De-Hyung Lee

**Affiliations:** 1 Department of Neurology, St Josef-Hospital, Ruhr-University Bochum, Bochum, Germany

## Abstract

Multiple sclerosis (MS) is the most common neurologic disease of young adults. In the recent years, our understanding on disease pathomechanisms has considerably improved and new therapies have emerged. Yet a cure for this devastating disorder is still a far cry away and human resources on *ex vivo *specimens are limited. More than 70 years after its first description, experimental autoimmune encephalomyelitis (EAE) remains an important tool to understand concepts of T cell mediated autoimmunity as well as the roles of the innate and the humoral immune systems. Some EAE models also well reflect mechanisms of tissue damage including demyelination, axonal injury and also cortical changes. A limitation of the classical EAE model is a neglect of CD8 T cell mediated immune mechanisms. Moreover, well characterized models for primary progressive MS or demyelination patterns involving primary oligodendrocyte dystrophy are still not available. Yet many current therapeutic concepts including glatiramer acetate or natalizumab stem from their successful first application in EAE models. New strategies include the widespread use of conditional knockout mice to understand the cell-type specific function of single genes, innovative approaches to establish models on the roles of B cells and CD8 T cells as well as on the relation of inflammation to primary degeneration. In summary, EAE models continue to play an important role in neuroimmunology thereby also stimulating research in other fields of the neurosciences and immunobiology.

## Classical EAE models: an overview

The model of experimental autoimmune encephalomyelitis (EAE) stems back from an attempt to understand the pathogenesis of post vaccinal encephalomyelitis, e.g., after rabies vaccination during the 1920s and 1930s (for overview see Gold et al., 2006). At that time, first immunization experiments with repeated inoculations of human spinal cord homogenate in rabbits and rhesus monkeys resulted in the first models of experimentally induced encephalomyelitis. Over the next 70-80 years, EAE was studied in many different species, including primates and rodents. Improved protocols included the application of a mineral oil-based adjuvant (Freund's adjuvant), and later on the addition of heat-inactivated mycobacteria (complete Freund's adjuvant, CFA) that facilitate disease induction after a single inoculation. Some models, especially in mice, also require the intraperitoneal injection of pertussis toxin which may deplete regulatory T cells and thus enhance autoimmune reactions [1].

After World War II, EAE models in rats and guinea pigs dominated neuroimmunological research for many decades. In these species, active immunization used either spinal cord homogenate, myelin or, as first purified antigen in the 1960s, myelin basic protein (MBP) in CFA. Such protocols resulted in a very reproducible disease with an onset 9-12 days after immunization and an acute monophasic or chronic relapsing-remitting/progressive disease course depending on the species or rodent strain [2]. Moreover, EAE can be induced not only by active immunization, but also passively by adoptive transfer of antigen specific T cells (AT-EAE). This was shown after in vitro propagation of MBP specific T cell lines and subsequent transfer of cells in mice [3]. In the Lewis rat strain, this regimen results in disease onset 3-4 days after cell transfer, followed by disease maximum 1-2 days later and a subsequent complete remission within the next few days. The histopathology of this model is characterized by a purely inflammatory pattern with minimal central nervous system (CNS) demyelination, thus rather reflecting acute disseminated encephalomyelitis (ADEM). Yet MBP-EAE provides an excellent paradigm to investigate T cell function and regulation in autoimmune neuroinflammation and nicely underpins the autoimmune origin of MS.

The induction of EAE is not restricted to myelin antigens, as the disease can also be elicited by astroglial antigens like S-100 or glial fibrillary acidic protein (for review see [4]) and neuronal antigens including Ma [5] or amyloid precursor protein (APP, [6]). Similarly, AT-EAE can also be induced by adoptive transfer of T cells specific for astroglial and neuronal antigens (reviewed in [7]).

Over the last 20-30 years, further encephalitogenic myelin antigens were identified, the first of which was proteolipid protein (PLP). Other myelin autoantigens in rodents include myelin oligodendrocyte glycoprotein (MOG; [8], myelin associated glycoprotein (MAG), 2', 3'-cyclic nucleotide 3'-phosphodiesterase (CNPase), oligodendrocyte (OL)-specific protein, and myelin-associated oligodendrocyte basic protein. The most recent studies were performed in inbred mouse strains in which immunodominant and encephalitogenic T cell epitopes, like PLP aa139-151 or MOG aa35-55, were also identified. Some of the mentioned myelin proteins are components not only of CNS, but also peripheral nervous system myelin. Thus immunization with e.g. MPB or PLP theoretically can also result in radiculitis or neuritis [9]. Importantly, not only the susceptibility to disease but also the pathology and clinical course are determined by both genetic factors and the specific immunogen/adjuvant used, resulting in established combinations of autoantigen and inbred mouse strain, e.g. PLP139-151 EAE in SJL mice. In some cases, tolerance to single autoantigens in non-susceptible strains can be overcome by specific protocols including booster immunization [10].

A milestone in multiple sclerosis (MS) research was marked by the characterization of MOG as an autoantigen. Although only a minor component of CNS myelin, MOG is part of the outer myelin layer thus being of special interest as target of a first autoimmune attack before antigen spreading may occur. Immunization with MOG induces not only encephalitogenic T cells, but also a demyelinating autoantibody response in susceptible species. Moreover, demyelinating anti-MOG antibodies enhance disease severity causing extensive demyelination after T cell-mediated CNS inflammation in rat and primate EAE models [11,12]. Such antibodies to native MOG are also found in MS patients and mediate demyelination after transfer into EAE rats [13]. At first, immunization with MOG in rodents was mainly investigated in different rat strains [14]. Studies in the DA rat were able to reproduce a range of pathological and clinical phenotypes mimicking the spectrum of MS [15]. Induction of MOG-EAE in Brown Norway rats led to a Devic like disease (at least in clinical terms) with eosinophilic infiltrates also suggesting the involvement of T helper (Th2) cells [16]. In congenic Lewis rats, the contribution of genetic and environmental factors to disease susceptibility and modulation of target tissue responses were characterized. In contrast, immunization of marmoset monkeys with CNS tissue homogenates or recombinant MOG provided a disease model closely mimicking humoral disease patterns of MS in a species that is phylogenetically very close to man [17,18]. Like in rats, pathogenic mechanisms include the contributions of encephalitogenic T cells and demyelinating autoantibodies [19]. While the investigation of marmoset EAE is somewhat limited by ethical issues as well as a fulminant disease course and lack of suitable markers in this species, this model may be of particular interest for pre-clinical treatment studies and imaging approaches.

In mice, MOG protein or MOG35-55 peptide can induce EAE in C57BL/6 mice or Biozzi ABH mice resulting in a relapsing-remitting or chronic disease course. In the C57BL/6 mouse strain, the H-2b MHC haplotype governs the ability to induce encephalitogenicity and to develop demyelinating autoantibodies in response to mouse or rat MOG. Transfer of these demyelinating anti-MOG antibodies also enhances demyelination and exacerbates disease severity in mouse models of EAE [8]. Over the last 15 years, the avenue of genetically engineered mice has greatly promoted the investigation of EAE pathomechanisms, particularly after backcrossing strains on the C57BL/6 background. Today, MOG-EAE in the C57BL/6 mouse is one of the most widely used models in neuroimmunological research with over 100 publications in recent years.

In summary, EAE models mimic many of the clinical, neuropathological and immunological aspects of MS but none is able to reproduce the whole spectrum of the human disease. In particular, many of the classical EAE models mainly affect the spinal cord (and not the brain) and are especially based on CD4 positive T cells, while the recently recognized importance of CD8 positive T cells in autoimmune inflammation of the CNS remains largely scotomized. So far, a model for the primary progressive course of the disease is still lacking and induction of EAE results in extensive tissue damage (including axons and neurons) rather than in primary demyelination. Nevertheless, the data from EAE studies still provide the most important cue for the notion of MS as an autoimmune disease where myelin specific cells initiate an inflammatory reaction in the CNS which ultimately leads to demyelination, gliosis and axonal injury.

## EAE models contribute to unveiling the immunopathogenesis of MS

Research in EAE first focused on the adaptive immune system including T and B cell responses. Recently, components of the innate immune system, in particular macrophages, microglia, Toll-like receptors and even mast cells, have also been recognized as important parts of disease pathogenesis [20,21]. In particular, the roles of vessel-associated dendritic cells for the re-activation of T cells in situ and of microglia for the development and maintenance of inflammatory CNS lesions have been highlighted [22,23]. Another recent focus is on B lymphocytes. Besides production of demyelinating antibodies after differentiation into plasma cells (see also above), B cells may have a co-stimulatory function for T cell effectors, but can also play a regulatory role [24,25].

The pivotal role of T lymphocytes in disease induction was confirmed by adoptive transfer of autoantigen specific T cells [26]. Later on, the importance of antigen-presenting cells (APC) governing T cell responses was recognized. Professional APC, like dendritic cells (DC), are characterized by a complete repertoire of co-stimulatory molecules like CD28/B7, inducible T cell co-stimulator (ICOS)/ICOS-L, and programmed death (PD)1/PD-1/2L thus enabling them to fully activate naive T cells, but also to regulate this activation (for overview see [27]. In contrast, non-professional APC, like macrophages, microglia or astrocytes, can upregulate the expression of immune molecules upon stimulation that facilitate activation only of memory, but not naive T cells. Recently, the special importance of perivascular DC for the reactivation of antigen specific T cells and their entry into the target organ was highlighted and DC may also present a valuable target for therapies as shown after intracerebral injection [28].

During T cell activation, the production of cytokines and chemokines influences the local microenvironment and finally determines the functional outcome of the immune response. In a classical paradigm, IFN-γ results in the differentiation of naive CD4 positive T cells into a "proinflammatory Th1" effector T cell subset which may initiate autoimmunity in the CNS [29]. In contrast, the "Th2" cytokines interleukin (IL)-4, IL-5, or IL-13 induce the differentiation of naive T cells into "Th2" T cells which are able to counterbalance the inflammatory reaction in the CNS. Only recently, the importance of IL-23 and not IL-12 for perpetuation of the immune response in EAE was recognized. This concept was further refined by the characterization of IL-17 producing "Th17" cells which seem to play a role for EAE induction in addition to "Th1" cells. While this concept was first established in rodents, a role for Th17 was recently also described in humans in vitro and in situ [30,31]. The generation of Th17 cells requires the presence of the "proinflammatory" cytokine IL-6 in concert with the "regulatory" cytokine transforming growth factor (TGF)-β, whereas IL-23 is involved in their maintenance [32]. An alternative pathway for the differentiation of Th17 cells may be induced by IL-21 [33]. In fact, cytokine regulation may involve complex networks rather than linear pathways. This notion is further corroborated by the involvement of the IL-18 receptor pathway in the generation of IL-17 responses [34]. Moreover, some T cell clones may be capable of producing both interferon gamma and IL-17, while IL-22 seems to be more specific for Th17 cells [35]. Some studies point at the importance of Th17 cells as part of the first wave of T cell infiltration into the CNS which may be mediated by specific chemokines and their receptors like CCR6 and CCL20 [36]. While IL-17 promotes autoimmunity by triggering a positive-feedback loop via interleukin-6 induction [37], other reports also found that Th17 cells are not in all cases necessary for the propagation of EAE [38]. Some recent data rather point at the importance of the transcription factor T-bet for governing encephalitogenicity of myelin-specific T cells rather than pathway specific cytokines such as IFN-γ or IL-17 [39]. Finally, Th9 cells entered the stage as most recent effector T cells. In vitro, this population is generated by a combination of IL-4 and TGF-β [40]. Besides IL-9, these cells also produce IL-10 and contribute to tissue inflammation like colitis or, as shown recently, also autoimmune inflammation of the CNS [41]. A further level of complexity was introduced by the identification of further leukocyte subtypes which can be involved in disease pathogenesis. In some models, pathogenic neuroantigen-specific Th2 T cell responses or neutrophilic as well as eosinophilic granulocytes as effector cells may be involved in tissue damage [42,43]. Moreover, mast cells produce IL-9 and thus may play a role in regulating T cell tolerance and in modulating the course of EAE [21,44].

The invasion of all these immune cells into the CNS requires transmigration over the blood brain barrier. Here, a complex molecular interaction takes place between adhesion molecules on the surface of immune cells and the cerebral endothelial cells forming the blood brain barrier [45,46]. These molecular interactions have been extensively studied in the 1990s. In particular, the interaction between the integrins very late antigen (VLA) 4 and leukocyte function associated antigen (LFA) 1 with their immunoglobulin-like counter-ligands vascular cell adhesion molecule (VCAM) 1 and intercellular adhesion molecule (ICAM) 1 was shown to be essential for T cell adhesion and migration into the central nervous system [47,48]. Recently, proteomic based approaches defined an important function for the activated leukocyte cell adhesion molecule (ALCAM) in the recruitment of leukocytes into the brain [49]. Meanwhile, many studies point to a role of the brain endothelium not only as barrier, but also as active immunomodulator involved in DC polarization and Th17 signaling pathways [50].

While Th17 cells promote autoimmune tissue inflammation, several regulatory cell types contribute to the delicate balance between autoimmunity and immunoregulation. In EAE, a regulatory role was first established for CD8 positive effector cells [51] and later also natural killer (NK)/NK-T cells [52]. Most recent work indicated that particularly regulatory T cells (Treg) characterized by the presence of the forkhead/winged helix transcription factor family member FoxP3 are necessary and sufficient to prevent autoimmunity both in rodents and in man. Interestingly, Th17 cells and FoxP3 positive Treg seem to be dichotomously regulated by TGF-β, which induces FoxP3 in naive T cells, but together with IL-6 drives the generation of Th17 cells [32]. In rat EAE, polyclonal expansion of Treg prevents transmigration of encephalitogenic T cells into the CNS [53]. In a murine model, naturally occurring FoxP3 positive Treg expand in the peripheral immune organs and also accumulate in the central nervous system (CNS), but then do not prevent the onset of EAE [54]. Thus, therapeutic approaches strengthening regulatory mechanisms may be more promising when additional regimens control tissue inflammation in parallel.

## Histopathology of EAE reflects many aspects of MS

Classical histopathological changes in MS lesions comprise perivascular inflammation, demyelination and gliosis in the white matter. Over the past 10 years, this concept has been refined and axonal injury, cortical demyelination as well as neuronal changes have come into the focus of interest. In acute demyelinating MS lesions, distinct patterns have been described pointing to patient-specific mechanisms of demyelination. In this classification, pattern I is characterized by the presence of T cells and macrophages and pattern II by antibody and complement deposition. In contrast, patterns III and IV are mediated by primary OL damage. In pattern III, an ischemic mechanism leading to loss of MAG, but not of MOG immunoreactivity, was postulated [55]. So far, EAE models predominantly reflect patterns I and II. In particular, antibody and complement deposition are detected in MOG-EAE lesions of marmosets and rats which are reminiscent of pattern II pathology [56]. In contrast, MOG-EAE in the C57BL/6 mouse results in less well demarcated demyelination which, after immunization with the T cell epitope MOG 35-55, is independent of humoral immune responses as revealed in μMT mice lacking mature B cells [57] and in models with deficiency for the complement component C3 or the C5a receptor [58,59]. Although the exact mode of demyelination in this model remains to be determined, myelin damage seems to be critically dependent on the presence of macrophages and possibly also tumor necrosis factor (TNF)-α. To evoke a significant B cell response in C57BL/6 mice, immunization with whole recombinant MOG protein is preferred. Studies on the role of antibodies may also involve co-transfer approaches. Here, a role for complement activation, but not FcRγ receptors on phagocytes, could be delineated for antibody mediated demyelination [60].

To a large extent, inflammatory demyelination in MOG-EAE is paralleled by apoptotic loss of OL in the lesions [61]. Thus, oligodendroglial repopulation may constitute an important prerequisite for myelin repair. Indeed, OL precursor cells have been identified in the rodent and human adult CNS (for review see [62]). In different experimental models of demyelination including EAE, oligodendroglial cells are recruited, proliferate and can differentiate into mature OL [63]. Here, glial growth factors as well as the re-expression of developmentally regulated oligodendroglial pathways, including expression of potassium channels like Kv1.4, may be relevant [64]. Yet, in murine MOG-EAE lesions, repair is largely incomplete resulting in an accumulation of lesions over time which correlates with persistent disability of mice. Thus, mechanisms of remyelination are preferably studied in toxic models of demyelination including the application of cuprizone, lysolecithine or ethidium bromide (for review see [65]).

In recent years, further research focused on the presence of grey matter demyelination in the cortex. Although this specific feature is not predominant in murine MOG-EAE, cortical demyelination was well characterized in a focal model of rat MOG-EAE [66] and in some Lewis rat strains (LEW.1AR1 and LEW.1W strains, [67]).

With the avenue of confocal and magnetic resonance imaging techniques, the importance of axonal damage re-entered the stage in neuroimmunological research [68]. In rat MOG-EAE models, patterns of axonal damage and, in some settings, also neuronal injury, were characterized [69,70]. After immunization with recombinant MOG, axonal damage was observed in the white matter of marmoset monkeys [17] and, in focal EAE models, also in the cortex of susceptible rat strains [66]. Investigation of axonal damage in chronic rat EAE and murine PLP-EAE underscored the concept that loss of axons may in particular correlate with permanent disability [71]. In murine MOG-EAE, axonal loss occurred early in lesions of white matter, but also in the grey matter of the spinal cord. At the later disease stages, disseminated axonal loss including the normal appearing white matter correlated with persistent disability [72]. Further studies in murine models were mainly performed in genetically engineered mice. Mice with a deficiency of the axonoprotective neurotrophic cytokine ciliary neurotrophic factor (CNTF) exhibited a more severe course of MOG-EAE with enhanced myelin and axonal pathology [61]. In a Thy1-GFP transgenic mouse model, patterns of axonal damage were directly characterized by fluorescent labelling of axon tracts in the spinal cord [73]. A focal EAE model in rats also allows the investigation of axonal remodelling in defined tracts [74]. In the near future, an elegant combination of these approaches in combination with 2-photon microscopy will be feasible in living animals [75]. Different mechanisms contribute to axonal damage during autoimmune demyelination (for overview see [76]). Among others, candidates include TNF-α mediated cytotoxicity, Fas-FasL interaction or glutamate excitotoxicity. Moreover, interaction of cytotoxic CD8 positive T cells with up-regulated major histocompatibility complex (MHC)-I on axons may play a role [77] although mice lacking MHC-I exhibit significant amounts of APP positive axons [78]. In several recent studies, altered expression of ion channels were implicated in axonal injury which may involve the sodoim channels Nav 1.2 or Nav1.6 [72] or the Ca^2+ ^and Na^+ ^permeable acid-sensing ion channel-1 [79]. Finally, EAE models allow investigation not only of axonal injury, but also of recovery and axonoprotection [80]. Recent studies focused on the role of the wlds fusion protein as an endogenous axonal protection mechanism [81] while some earlier studies in rodents characterized neuroprotective treatment approaches, like blocking of glutamate receptors [82]. Moreover, blocking of sodium channels may provide an attractive new therapeutic option (among others, see [83]), although expression of these channels in the immune system may somewhat limit these approaches and first studies in MS were reportedly not successful.

## The value of experimental models for imaging studies and innovative therapies

EAE models may not only serve for the characterization of immunological and neurobiological mechanisms, but can also be valuable for the pre-clinical testing of new diagnostic or treatment modalities. Different magnetic resonance imaging (MRI) techniques have been employed to depict EAE lesions in rat brains, including contrast enhancement with gadolinium or ultra small iron particles [84], which allow macrophage tracking [85]. While most investigations focused on MBP-EAE models, some recent studies also investigated MOG-EAE in rats [86], marmosets [87] and also murine EAE models [88]. Yet imaging of spinal cord is still not satisfactory with routine protocols, and is only feasible with high-resolution techniques [89]. Advanced MR protocols may also include diffusion tensor imaging which is able to detect selective vulnerability of cerebral white matter in murine EAE [90]. More sophisticated protocols even allow imaging on a molecular level. These techniques include ultrasound based approaches which are able to depict and quantify adhesion molecules in living EAE rats with an axial resolution in the μm range [91]. Similarly, antibody-conjugated microparticles carrying iron oxide can provide quantifiable MRI contrast effects that delineate the architecture of activated cerebral blood vessels [92].

Regarding treatment approaches, many established present-day MS therapies like glatiramer acetate as well as natalizumab stem from their first successful application in EAE models. Yet a plethora of new therapeutic approaches are effective in animal models, but have failed in human trials. Well known failures include anti-TNF therapies in MS patients [93] as well as the application of superagonistic anti-CD28 antibodies in healthy volunteers [94]. In case of the anti-TNF studies, negative results in patients were better understood when data from knockout mouse models became available which revealed a beneficial role for TNF-α in apoptotic clearance of T cells from inflammatory lesions [95]. Even though a thorough characterization of CD28 superagonistic antibody therapy in animal models of both peripheral and CNS inflammation [96,97] as well as later on also with live imaging of immune organs [98] was performed, this did not foresee the multiple cytokine release syndrome leading to severe side effects in a human phase I study. These failures call for the highest possible awareness with new therapies in human beings. EAE experiments cannot substitute for, but are rather complementary to, human clinical studies, and maximum safety standards during the first application of new compounds in patients must always apply. Yet, if interpreted correctly and chosen with care, EAE models can provide useful information, and sometimes may avoid complications which delay drug development or pose a threat to patients. In particular, EAE models may help to elucidate mechanisms of action for new MS therapies. One example is fumarate (FAE) therapy in MS, currently being tested in a human phase III trial. In the human skin disease psoriasis, FAE was shown to be effective via a Th1/Th2 shift. Yet, in EAE, FAE seems to exert effects on macrophages [99] and possibly also to harbour neuroprotective effects. Since other regenerative treatment approaches for MS are still a long way ahead, the best neuroprotective approach still consists in effective immunotherapy. This can be convincingly shown in treatment of rat MOG-EAE with liposomal steroids which effectively deplete phagocytes, modulate T cells and at the same time preserve axons in inflammatory lesions [100].

## Future developments and new strategies

Future directions include EAE studies in conditional knockout mice to carve out the contribution of single molecules to the induction or effector phase of EAE. First studies used the Cre loxP system to conditionally delete genes involved in nuclear factor-κB (NF-κB) signalling pathways (EAE in CNS-restricted NEMO knockout mice, [101]). Together with the generation of bone marrow chimeras, these techniques provide powerful tools to dissect mechanisms during autoimmune inflammation of the CNS. Other innovative approaches include the immunization of Lewis rats with alpha synuclein leading to grey matter pathology which recently came into the focus of interest [102]. In the grey matter, also contactin2/TAG-1 mediated autoimmunity was recently characterized as contributing to cortical damage [103].

Moreover, the characterization of neurofascin as a novel autoantigen on axons at the node of Ranvier [104] and the identification of a protective role for the candidate astroglial autoantigen αβ crystallin in autoimmune demyelination [105] shed further light on non-myelin proteins as targets in disease pathogenesis. Further strategies are aiming at the implementation of models for aspects of MS which are not well mimicked in EAE paradigms so far. These efforts include studies on CD8 positive T cells which are a major component of the inflammatory infiltrate in chronic MS lesions [106]. In fact, several approaches in mice demonstrated that CD8 positive T lymphocytes also harbor an encephalitogenic potential [107-109] and are able to attack and transect MHC class I expressing axons in an antigen-specific manner. Recently, an elegant viral model studying cytotoxic T cells in lymphocytic choriomeningitis virus (LCMV) infection demonstrated that two related, but independently encountered, viral infections can lead to organ-specific immune disease without molecular mimicry or breaking immune tolerance [110].

CD8 T cell restricted autoimmune reactions can also be studied after conditional expression of MHC-I as shown in the muscle [111] or in double transgenic mice with simultaneous an OL sequestered antigen like ovalbumin or influenca hemagglutinin in the context of a respective MHC-I restricted T cell receptor [112,113]. In such settings, monoclonal antibodies specific for MHC class I molecules presenting a dominant autoantigen peptide may allow selective immunotherapy [114]. Such transgenic approaches were also used to study CD4 T cell and B cell mediated immune reactions. In genetically engineered mice exclusively harbouring an MBP specific T cell receptor (TCR), EAE develops spontaneously with a high incidence [115]. In a similar approach, MOG-TCR transgenic mice suffer from spontaneous autoimmune optic neuritis to some degree [116]. In contrast, double transgenic mice with a MOG specific TCR and MOG-specific B cells spontaneously develop a Devic like disease pattern with lesions exclusively in the optic nerve and spinal cord, thus nicely underscoring the cooperation of B cells and T cells in the development of autoimmune inflammation [117,118]. These spontaneously occurring models do not require artificial immunization protocols with adjuvant or pertussis toxin and are therefore of special value for neuroimmunological studies. In this MOG TCR transgenic model, autoreactive T cells harbor a bi-specificity and recognize their MOG epitope, but also neurofilament M as self-antigen. Such cumulative responses against several autoantigens by one autoreactive T cell clone may also contribute to pathogenicity in MS [119]. Whilst most of these spontaneously occurring models lead to a chronic disease course, a recently developed model with a transgenic MOG92-106 specific TCR in the context of an I-A(s) background was also able to spontaneously mimic relapsing remitting disease course [120]. Further elegant tools are humanized transgenic mice which can be used as tools to study human T cell receptors (TCR) in experimental models. This was first shown in a transgenic mouse model for an MHC-II restricted TCR specific for MBP84-102 [121]. While the incidence of spontaneous EAE in this model is low, immunization with a microbial peptide results in induction of disease, which may be due to structural mimicry of a binding hotspot shared by self and microbial antigens rather than to a degenerate TCR recognition [122]. Recently, such concepts were also analyzed in a transgenic model harboring a myelin specific T cell receptor of a CD8 T cell clone from a MS patient and the respective MHC class I allele. In this model, CD 8 positive T cells can exert disease promoting or protective functions, depending on the MHC class I context [123].

So far, EAE models cover the spectrum of demyelination patterns I and II, but they do not well reflect patterns III and IV with primary OL dystrophy. Yet, this obstacle may be overcome by the analysis of mutants with primary demyelination due to a genetic defect. This was recently suggested in a study of mice with peroxisome-deficient OL. Here, a metabolic defect in myelin producing cells led to demyelination and subsequently also CNS inflammation with infiltration of B cells and activated CD8 positive T cells [124], similar to previous investigations in knockout models of PNS myelin [125].

Finally, all these data from EAE models may also promote knowledge on immune reactions in other neurologic diseases, like infectious disorders of the CNS [126] or post-stroke immunodepression [127].

## Competing interests

The authors declare that they have no competing interests.

## Authors' contributions

RAL and DHL performed the literature search and wrote the manuscript.
